# Discovering functional interaction patterns in protein-protein interaction networks

**DOI:** 10.1186/1471-2105-9-276

**Published:** 2008-06-11

**Authors:** Mehmet E Turanalp, Tolga Can

**Affiliations:** 1 Department of Computer Engineering, Selcuk University, Alaaddin Keykubat Kampusu, 42075 Selcuklu, Konya, Turkey; 2 Department of Computer Engineering, Middle East Technical University, Inonu Bulvari, 06531 Cankaya, Ankara, Turkey

## Abstract

**Background:**

In recent years, a considerable amount of research effort has been directed to the analysis of biological networks with the availability of genome-scale networks of genes and/or proteins of an increasing number of organisms. A protein-protein interaction (PPI) network is a particular biological network which represents physical interactions between pairs of proteins of an organism. Major research on PPI networks has focused on understanding the topological organization of PPI networks, evolution of PPI networks and identification of conserved subnetworks across different species, discovery of modules of interaction, use of PPI networks for functional annotation of uncharacterized proteins, and improvement of the accuracy of currently available networks.

**Results:**

In this article, we map known functional annotations of proteins onto a PPI network in order to identify frequently occurring interaction patterns in the functional space. We propose a new frequent pattern identification technique, PPISpan, adapted specifically for PPI networks from a well-known frequent subgraph identification method, gSpan. Existing module discovery techniques either look for specific clique-like highly interacting protein clusters or linear paths of interaction. However, our goal is different; instead of single clusters or pathways, we look for **recurring **functional interaction patterns in arbitrary topologies. We have applied PPISpan on PPI networks of *Saccharomyces cerevisiae *and identified a number of frequently occurring functional interaction patterns.

**Conclusion:**

With the help of PPISpan, recurring functional interaction patterns in an organism's PPI network can be identified. Such an analysis offers a new perspective on the modular organization of PPI networks. The complete list of identified functional interaction patterns is available at .

## Background

In the last few years, with the advances in high-throughput techniques, like yeast two-hybrid [1,2] and affinity purification coupled with mass spectrometry [3,4], the complete sets of interacting proteins of an increasing number of organisms have been identified [5]. In addition, probabilistic techniques that utilize indirect genomic evidence have provided increased genome coverage by predicting new interactions with multiple supporting evidence [6,7].

In parallel with the availability of genome-scale protein networks, various studies have been conducted to analyze these networks in order to understand their topological organization [8-10], identify conserved subnetworks across different species [11,12], discover modules of interaction [13-18], predict functions of uncharacterized proteins [19-21], and improve the accuracy of currently available networks [5,22-26]. In this study, we use available functional annotations of proteins in a PPI network and look for overrepresented patterns of interaction in the network. The patterns we look for are recurring **subgraphs **of arbitrary topologies. Similar studies, which aim to find frequent subnetworks in a larger network, have been conducted on gene regulatory networks [27,28] and chemical compound networks [29-31]. The discovery of frequent patterns in gene regulatory networks is shown to be biologically interesting by the early seminal work of Uri Alon's group [27,28]. They found small (3–4 node) but significant patterns, i.e., network motifs, in the transcription regulation network of *E. Coli *and provided biologically meaningful explanations for a number of those patterns. The network motifs they present have specific functions in determining gene expression, such as generating temporal expression profiles and governing the responses to fluctuating external signals. Alon *et al*., later, improved their algorithms for detecting motifs in networks with two or more types of interactions and applied them to an integrated dataset of protein-protein interactions and transcription regulation in *Saccharomyces cerevisiae *[28]. However, in that follow-up study, they again seek for gene regulatory patterns. Our work can be thought of as an adaptation of Alon's work on gene regulatory patterns to protein-protein interaction patterns.

There have been a number of studies on PPI networks for mining interaction patterns on a large scale [11,12,32-34]. Sharan *et al*. [11], Koyuturk *et al*. [34], and Hirsh and Sharan [12] analyzed PPI networks of several organisms and discovered conserved interaction patterns across species. The reported patterns correspond to specific biological processes common to the studied organisms. Oyama *et al*. [32] and Besemann *et al*. [33] used association rule mining techniques for finding interaction rules between protein pairs. To the best of our knowledge, PPI networks of individual organisms have not been mined for recurring interaction subgraphs of arbitrary topologies.

In a PPI network, an edge between two proteins indicates a physical association in the form of modification (e.g., phosphorylation), transport, or complex formation via physical binding [1]. In other words, subcomponents of a genome-scale PPI network may represent functional modules such as molecular complexes, signal transduction, or transport pathways. Similar to recurring regulatory patterns in gene regulatory networks, a functional interaction template may occur in different contexts in a modularly organized PPI network.

In order to find frequent functional interaction patterns in a PPI network, we first label the nodes of the network with functional categories using available functional annotations provided by databases such as Gene Ontology [35]. In other words, we project the functional annotation space onto the PPI network. In such a labeled network, recurring functional interaction patterns between different functional categories may emerge and provide biological insights into the functional organization of PPI networks. In this study, we use the *Molecular Function *hierarchy of the Gene Ontology annotations to assign functional categories to proteins in an interaction network. We focus on functional interaction patterns; therefore, *Cellular Component *and *Biological Process *ontologies are not considered in this study. We use the GO Slim subset [36] (see Table 1) of the molecular function terms which provides a broad overview of the functional categories in GO.

**Table 1:** GO Slim Molecular Function Terms for S. Cerevisiae

**Term ID**	**Definition**
GO:3674	molecular function unknown
GO:16787	hydrolase activity
GO:16740	transferase activity
GO:5515	protein binding
GO:3723	RNA binding
GO:5215	transporter activity
GO:5198	structural molecule activity
GO:30528	transcription regulator activity
GO:16491	oxidoreductase activity
GO:3677	DNA binding
GO:30234	enzyme regulator activity
GO:8233	peptidase activity
GO:16874	ligase activity
GO:4672	protein kinase activity
GO:16779	nucleotidyltransferase activity
GO:16829	lyase activity
GO:4386	helicase activity
GO:16853	isomerase activity
GO:45182	translation regulator activity
GO:4871	signal transducer activity
GO:4721	phosphoprotein phosphatase activity
GO:3774	motor activity

The 22 broad functional categories (including the term *molecular function unknown*) as given by the GO Slim database for the Molecular Function hierarchy of the Gene Ontology terms.

Two recent studies also map GO annotations on biological networks to find unknown and significant pathways. Cakmak and Ozsoyoglu [18] propose a supervised method for finding pathways across organisms. Using known pathways in databases such as KEGG [37], they learn functional templates representing these pathways. They use the templates to discover new pathways in the metabolic network of a new organism. However, this supervised technique is limited by the reference pathways and cannot be used to detect completely novel pathways. We propose an unsupervised method which looks for abundant functional interaction patterns in the PPI network of a target organism. In that sense, the patterns we discover are not specific pathways but higher level functional templates that recur in a number of contexts in the PPI network. Pandey *et al*. use GO terms to annotate regulatory and signaling pathways and find significantly recurring pathways in molecular interaction networks [38,39]. The software they have developed, NARADA, allows researchers to discover significantly overrepresented patterns of interaction in PPI or regulatory networks of any organism for any type of annotations. However, the proposed method can find linear pathways of size 2 to 5 and interaction patterns of different topologies are not sought. The method we propose in this article is able to find functional interaction patterns that may exhibit arbitrary topologies. Especially, since a PPI network contains non-linear subcomponents such as molecular complexes, the ability to discover interaction patterns of arbitrary topologies provides an increased coverage of overrepresented patterns. One may argue that molecular complexes are expressed as clique like highly interacting clusters in PPI networks and do not have interesting interaction topologies. However, molecular complexes of arbitrary topologies can indeed be formed. Recent studies on the structure of molecular complexes show that a small number of topological arrangements are favored in the space of all possible arrangements [40]. Hence, it may be biologically interesting to study the recurring molecular complex topologies in a PPI network [41]. Studying molecular complex topologies in a noisy PPI network is challenging and prone to produce false positive complex topologies. However, as PPI networks get more accurate and provide more genome coverage, such problems will cease to exist.

There are various algorithms developed for discovering frequent patterns in graphs [21,31,34,42-45]. The details of these methods are given in the Methods section.

In this article, we propose a new frequent pattern identification technique, PPISpan, for frequent pattern mining in functionally annotated PPI networks. Our goal in this study is not to discover novel complexes or pathways, which is studied extensively by many researchers [13-18]; instead, we try to discover **recurring **functional interaction patterns to understand whether such patterns are reused in different contexts in a PPI network. Our technique, PPISpan, is a modification of the gSpan algorithm [31] to better suit for PPI networks annotated with broad functional categories. We applied PPISpan on experimentally determined and predicted PPI networks of baker's yeast (*Saccharomyces cerevisiae*) labeled with molecular function GO annotations and identified a number of potentially interesting interaction patterns. The reported functional interaction patterns are abstract and cannot be verified by wet-lab experiments. But, in an effort to validate some of the discovered frequent functional interaction patterns, we compare their supporting embeddings with known molecular complexes and pathways. A supporting embedding of a functional interaction pattern is a specific instance of the functional pattern realized by certain proteins in the PPI network. We find non-overlapping embeddings using PPISpan.

## Results and Discussion

### Results

We implemented PPISpan in C++ and run all our experiments on a personal workstation with two Intel Xeon 2.66 GHz dual core CPUs and 4 GBs of memory. We searched for patterns on three of the PPI networks of *Saccharomyces cerevisiae *available in public databases: 1) DIP database which contains experimentally determined interactions [46], 2) STRING database which provides confidence weighted predicted interactions using multiple data sources, and 3) WI-PHI database which provides confidence weighted predicted interactions enriched for physical interactions. We labeled the nodes of the PPI network using the available GO Slim molecular functional annotations for yeast proteins (see Table 1). See the Methods section for details of the datasets used in our experiments.

We searched for frequent interaction patterns of support 15 or higher. We experimented with different values of minimum support threshold and conclude that the minimum support threshold value of 15 provides a reasonable number of frequent patterns in a reasonable running time. Table 2 shows the number of significant frequent patterns found in the three PPI networks. We do not report the patterns that include proteins annotated with the term *molecular function unknown*.

**Table 2:** The number of patterns found

**PPI Network**	**Number of Frequent Patterns**	**Number of Patterns with z-score > 2.3**
DIP	205	199
STRING	287	17
WI-PHI	378	321

The number of distinct frequent patterns that have at least 15 non-overlapping embeddings in the PPI networks. The number of patterns with a z-score greater than 2.3 is also shown in the last column.

PPISpan identified a total of 205 frequent interaction patterns with support >= 15 in the DIP network. 199 of the interaction patterns are significant with a z-score of > 2.3. The frequent interaction patterns cover 37.06% (1828 proteins) of the DIP network. For the STRING network, there are 287 frequent interaction patterns, only 17 of which have z-scores greater than 2.3. The frequent patterns cover 40.79% (1204 proteins) of the STRING network. We have identified 378 frequent patterns in the WI-PHI network, of which 321 are statistically significant. The frequent patterns cover a total of 1734 proteins of the WI-PHI network (37.27%). Although the embeddings of the reported patterns are non-overlapping, the patterns themselves may overlap provided that a pattern is not a subgraph of another pattern. Most of the patterns we found are trees. Star topology is the most abundant frequent pattern topology. Cycles are rare. This observation suggests that approximate but fast algorithms for tree pattern mining can be utilized to search for patterns in PPI networks to achieve near interactive response times.

In the next section, we validate a number of selected functional interaction patterns by comparing their supporting embeddings with known molecular complexes and pathways. Then, we present a number of functional interaction patterns that may be of interest to the reader.

#### Comparison with Known Molecular Complexes and Pathways

A genome-scale PPI network is composed of functional modules such as molecular complexes, signaling, and transport pathways. On the other hand, functional interaction patterns found by PPISpan are subgraphs with certain types of nodes that reoccur in a number of contexts in a PPI network. In this section, we try to interpret and validate some of the patterns using existing biological knowledge. We want to emphasize again that the goal of pattern finding is not discovering novel complexes or pathways. Our goal is to understand the underlying functional interaction mechanisms and whether such mechanisms are reused in different contexts in PPI networks.

A reasonable approach to analyze the discovered frequent interaction patterns is to compare their supporting embeddings with known molecular complexes and pathways. In our experimental setup, we compare the *proteins *(i.e., nodes) of supporting embeddings to a set of molecular complexes and pathways ignoring the edges that represent the interaction. Ideally, the topology of the interaction patterns should also be compared with molecular complex and pathway topologies. However, the molecular complex data we use do not provide the specific interactions between complex members and list only the proteins involved. Therefore, in this section, we ignore the topology of the frequent interaction patterns and treat the patterns as a set of proteins.

We collected molecular complexes from the MIPS complex catalogue database [47] and signaling, transport, and regulatory pathways from the KEGG database [37]. Discarding the complexes resulting from high-throughput experiments, we used the remaining high-quality set of 267 MIPS complexes as known molecular complexes. The KEGG pathways we used as known signaling and transport pathways are: ABC transporters, MAPK signaling pathway, phosphatidylinositol signaling system, SNARE interactions in vesicular transport pathway, and regulation of autophagy pathway.

We propose two measures in order to interpret and validate a frequent functional interaction pattern. The first measure is the average number of different complexes or pathways the embeddings of a frequent interaction pattern overlaps with. We name this measure as *cpcount*. The purpose of this measure is to speculate on the location of the interaction patterns, i.e., whether they are within or at the interfaces of complexes. The second measure, *cpoverlap *quantifies the overlap between proteins in an embedding and known complexes/pathways. The overlap measure for an embedding *e *is computed as the ratio of proteins in *e *that are members of known functional modules:

$$cpoverlap(e)=\frac{\left|\{p|p\in e\wedge p\text{ is identified in a known functional module}\}\right|}{\left|e\right|}$$

As we have stated in the beginning of this section, we disregard the interactions (i.e., edges) and instead focus on the set of proteins contained in an embedding.

A recurring functional interaction pattern is more likely to include protein interactions that occur within or at the interfaces of known functional modules such as complexes and pathways. So, a pattern should overlap with one or more complexes or pathways. However, the known set of complexes and pathways we collected are far from complete and cover only 1178 proteins (23.9%) of the DIP PPI network [46]. Therefore, not all the frequent patterns will overlap with known complexes and pathways. We performed a systematic analysis on all the frequent patterns. However, our experiments showed that the overlap with known complexes and pathways are not significantly different than random embeddings of similar topologies found by ignoring functional annotation labels of nodes (see Tables 3 and 4). We believe the main reason for this observation is that some of the observed patterns contain very general functional terms and hence the patterns are not specific enough in terms of function. In other words, for some of the observed patterns, the topology is more important than the underlying functional annotations, which makes them similar to the random embeddings. Therefore, in this section we validate a number of selected interaction patterns which are biologically more interesting.

**Table 3:** Comparison of all the patterns with random patterns in terms of overlap with MIPS complexes

**PPI Network**	***cpoverlap* of Frequent Patterns**		***cpoverlap *of Random Patterns**	
	
	Mean	Standard Deviation	Mean	Standard Deviation
DIP	0.729	0.111	0.718	0.049
STRING	0.835	0.056	0.731	0.036
WI-PHI	0.609	0.138	0.657	0.054

The average *cpoverlap *measure for all the patterns discovered in DIP, STRING, and WI-PHI networks compared to the average *cpoverlap *measure of random patterns of same topology with respect to the MIPS complexes.

**Table 4:** Comparison of all the patterns with random patterns in terms of overlap with transport and signaling pathways

**PPI Network**	***cpoverlap *of Frequent Patterns**		***cpoverlap *of Random Patterns**	
	
	Mean	Standard Deviation	Mean	Standard Deviation
DIP	0.982	0.050	0.959	0.013
STRING	0.979	0.033	0.991	0.014
WI-PHI	0.982	0.043	0.948	0.017

The average *cpoverlap *measure for all the patterns discovered in DIP, STRING, and WI-PHI networks compared to the average *cpoverlap *measure of random patterns of same topology with respect to the transport and signaling pathways.

**Figure 1:**
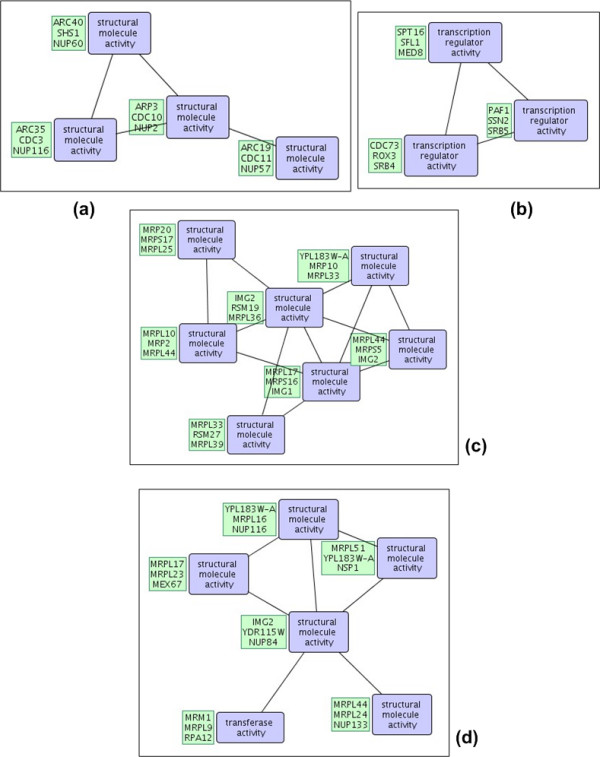
**Selected patterns from DIP and WI-PHI networks**. Two selected frequent interaction patterns in the DIP network with highest *cpoverlap *values with respect to MIPS complexes are shown in (a) and (b). The patterns in (a) and (b) correspond to patterns #1 and #2 in the text and in Figures 3 and 4. Another two selected frequent interaction patterns in the WI-PHI network with highest *cpoverlap *values are shown in (c) and (d). The patterns in (c) and (d) correspond to patterns #5 an #6 in the text and in Figures 3 and 4. The first three embeddings of the patterns are shown in green boxes. The blue boxes show the molecular function annotations of the nodes.

**Figure 2:**
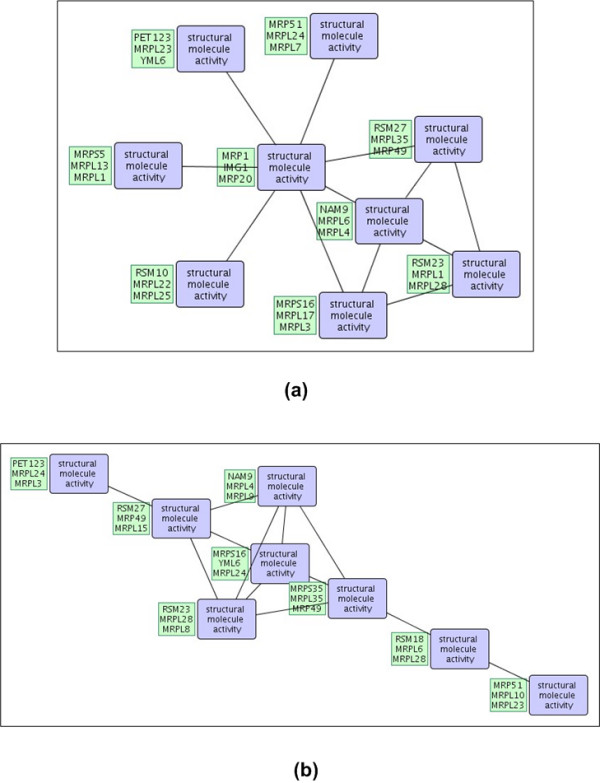
**Selected patterns from the STRING network**. Two selected frequent interaction patterns in the STRING network with highest *cpoverlap *values with respect to MIPS complexes are shown in (a) and (b). The patterns in (a) and (b) correspond to patterns #3 and #4 in the text and in Figures 3 and 4. The first three embeddings of the patterns are shown in green boxes. The blue boxes show the molecular function annotations of the nodes.

**Figure 3:**
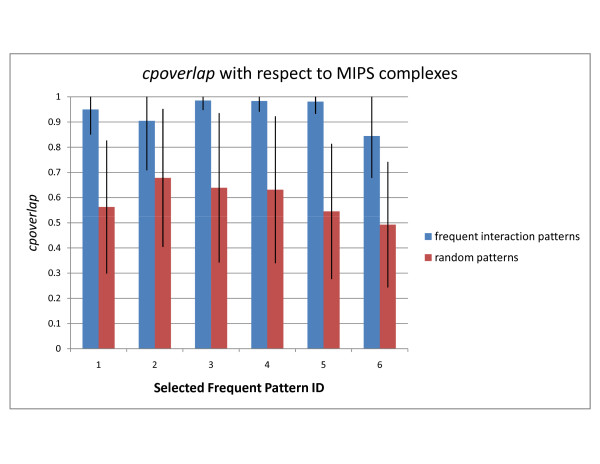
**cpoverlap of selected patterns with respect to MIPS complexes**. The average *cpoverlap *values of the selected frequent patterns and random embeddings of same topology are shown. *cpoverlap *of an embedding is the ratio of proteins that are members of known functional modules. The values are computed with respect to MIPS complexes. Each embedding of a frequent pattern may have a different *cpoverlap *value. The standard deviation of the *cpoverlap *values of the embeddings of a pattern is indicated as error bars with one standard deviation in each direction. Pattern IDs represent the selected patterns shown in Figures 1 and 2.

**Figure 4:**
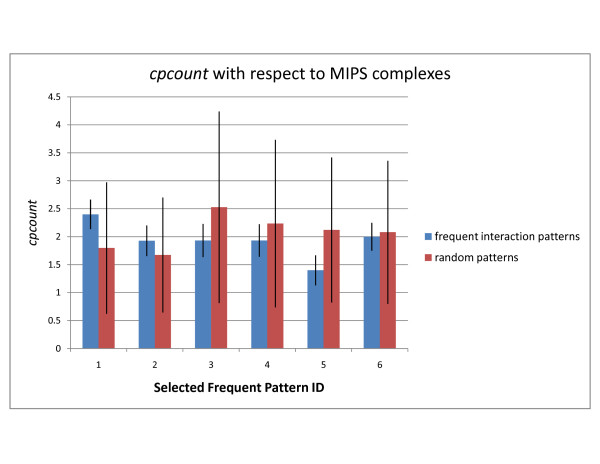
**cpcount of selected patterns with respect to MIPS complexes**. The average *cpcount *values of the selected frequent patterns and random embeddings of same topology are shown. *cpcount *is given as the number of different complexes or pathways an embedding of a frequent interaction pattern overlaps with. The values are computed with respect to MIPS complexes. Each embedding of a frequent pattern may have a different *cpcount *value. The standard deviation of the *cpcount *values of the embeddings of a pattern is indicated as error bars with one standard deviation in each direction. Pattern IDs represent the selected patterns shown in Figures 1 and 2.

Below, we analyze the top-2 patterns discovered in the DIP, STRING, and WI-PHI networks with highest *cpoverlap *values and show that their overlap is significant by comparison to random embeddings of same topology. Selected patterns are shown in Figures 1 and 2. Random embeddings are found using PPISpan and ignoring the molecular function annotations. Figures 3 and 4 show the *cpoverlap *and the *cpcount *values computed for the six selected patterns with respect to MIPS complexes, respectively. Figure 3 shows that all *cpoverlap *values for the frequent functional interaction patterns are significantly greater than that of random embeddings of same topology (p-value = 0). The embeddings of the frequent patterns discovered in the DIP network, i.e., patterns #1 and #2, overlaps with 2.4 and 1.9 MIPS complexes on the average (Figure 4). *cpcount *of pattern #1 is significantly greater than the *cpcount *of random patterns with a p-value of 0.0372. However, the difference between pattern #2 and it random embeddings is not significant (p-value = 0.3268). The *cpcount*s of the patterns found in STRING and WI-PHI networks are smaller than the corresponding *cpcounts *of random embeddings. Nevertheless, we can observe that the functional interaction patterns overlap with 2 MIPS complexes on the average, suggesting that a functional interaction pattern is more likely to exist at the interface of two complexes. Indeed, the sample embeddings of the STRING and WI-PHI patterns shown in Figures 1 and 2, show interactions between large and small subunits of the mitochondrial ribosomal protein (MRPS* and MRPL* gene ids).

**Figure 5:**
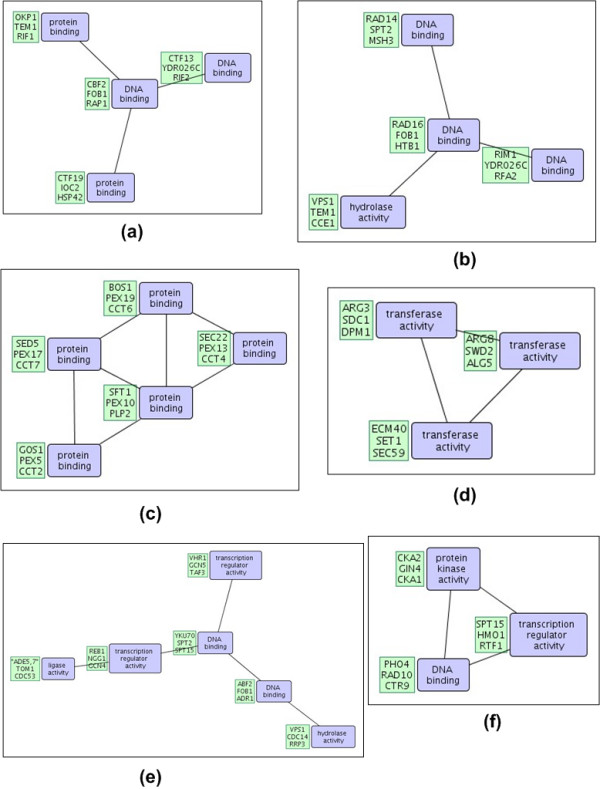
**Selected patterns from DIP, STRING, and WI-PHI networks**. Six selected frequent interaction patterns from the DIP, STRING, and WI-PHI networks with highest *cpoverlap *values with respect to KEGG pathways are shown. The patterns in (a) through (f) correspond to patterns #7 through #12 in the text and in Figures 6 and 7. The first three embeddings of the patterns are shown in green boxes. The blue boxes show the molecular function annotations of the nodes.

**Figure 6:**
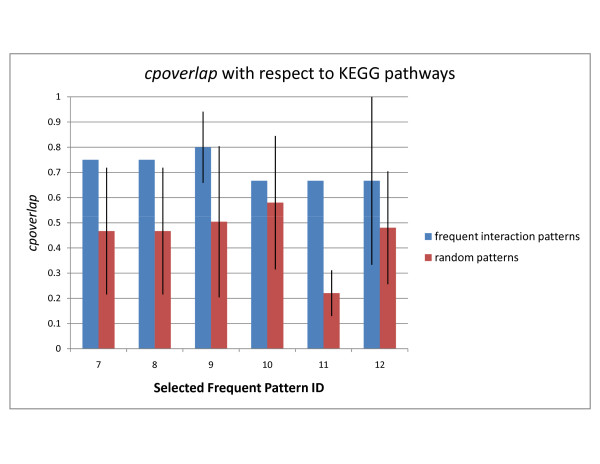
**cpoverlap of selected patterns with respect to transport and signaling pathways**. The average *cpoverlap *values of the selected frequent patterns and random embeddings of same topology are shown. *cpoverlap *of an embedding is the ratio of proteins that are members of known functional modules. The values are computed with respect to KEGG pathways. Each embedding of a frequent pattern may have a different *cpoverlap *value. The standard deviation of the *cpoverlap *values of the embeddings of a pattern is indicated as error bars with one standard deviation in each direction. Pattern IDs represent the selected patterns shown in Figure 5.

**Figure 7:**
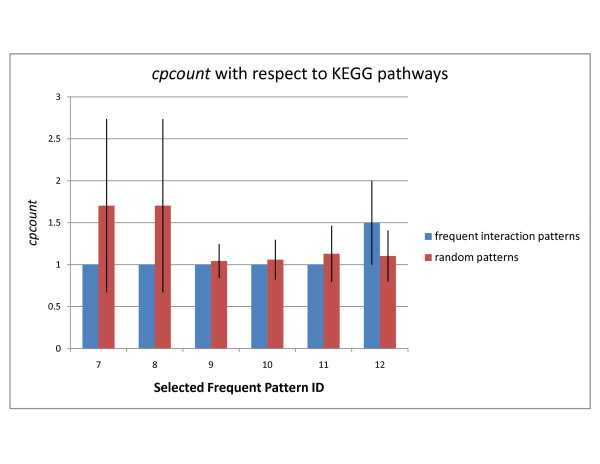
**cpcount of selected patterns with respect to transport and signaling pathways**. The average *cpcount *values of the selected frequent patterns and random embeddings of same topology are shown. *cpcount *is given as the number of different complexes or pathways an embedding of a frequent interaction pattern overlaps with. The values are computed with respect to KEGG pathways. Each embedding of a frequent pattern may have a different *cpcount *value. The standard deviation of the *cpcount *values of the embeddings of a pattern is indicated as error bars with one standard deviation in each direction. Pattern IDs represent the selected patterns shown in Figure 5.

Figure 5 shows the six selected patterns from the three PPI networks which exhibit highest *cpoverlap *values when compared against the KEGG pathways. All of the selected patterns in Figure 5 are related to transcription regulation. Figures 6 and 7 show the *cpoverlap *and the *cpcount *values computed for the six selected patterns in Figure 5 with respect to KEGG transport and signaling pathways, respectively. The results are quite different from MIPS complex overlap results. This is mostly because the number of pathways (5) used in the analysis is significantly smaller compared to the number of MIPS complexes (267). The transport and signaling pathways cover a very small region of the PPI networks. However, Figure 6 shows that the *cpoverlap *values of the selected patterns are again significantly higher compared to the *cpoverlap *values of the random embeddings except pattern #10 which does not show a significant difference (p-value = 0.4165). The average number of KEGG pathways contained in an embedding is around 1 (Figure 7). However, the relatively small number of embeddings that overlap with the known pathways prevents us from drawing conclusions about average overlap count.

In summary, our validation efforts show that the embeddings of some of the discovered interaction patterns significantly overlap with known molecular complexes and pathways and the functional interaction patterns are mostly at the interface of two of molecular complexes and within single pathways.

#### Some Interesting Functional Interaction Patterns

**Figure 8:**
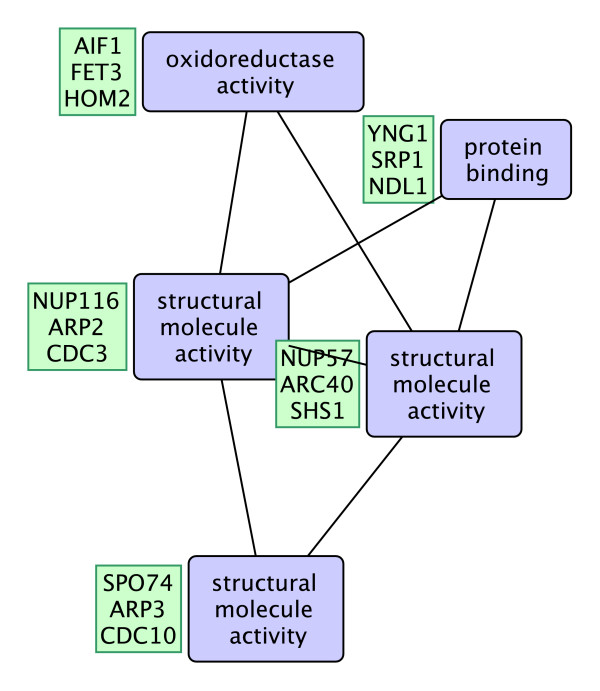
**A frequent functional interaction pattern in the DIP network**. A frequent function interaction pattern in DIP network. The pattern includes 5 proteins and has 15 non-overlapping occurrences in the network (z-score = 7.4).

In this section, we present a number of functional interaction patterns that may be interesting for biologists. Figure 8 shows a functional interaction pattern with cycles discovered in the DIP network. The pattern includes 5 proteins and has 15 non-overlapping occurrences with a z-score of 7.4. The pattern contains 3 structural molecule activity terms, one protein binding term, and an oxidoreductase activity term. Three of the fifteen embeddings of this term is given in green boxes to the left of the nodes. The activities represented in these patterns are central to many of the cell activities therefore it is not surprising to see that these patterns are occurring frequently in the PPI network of yeast.

**Figure 9:**
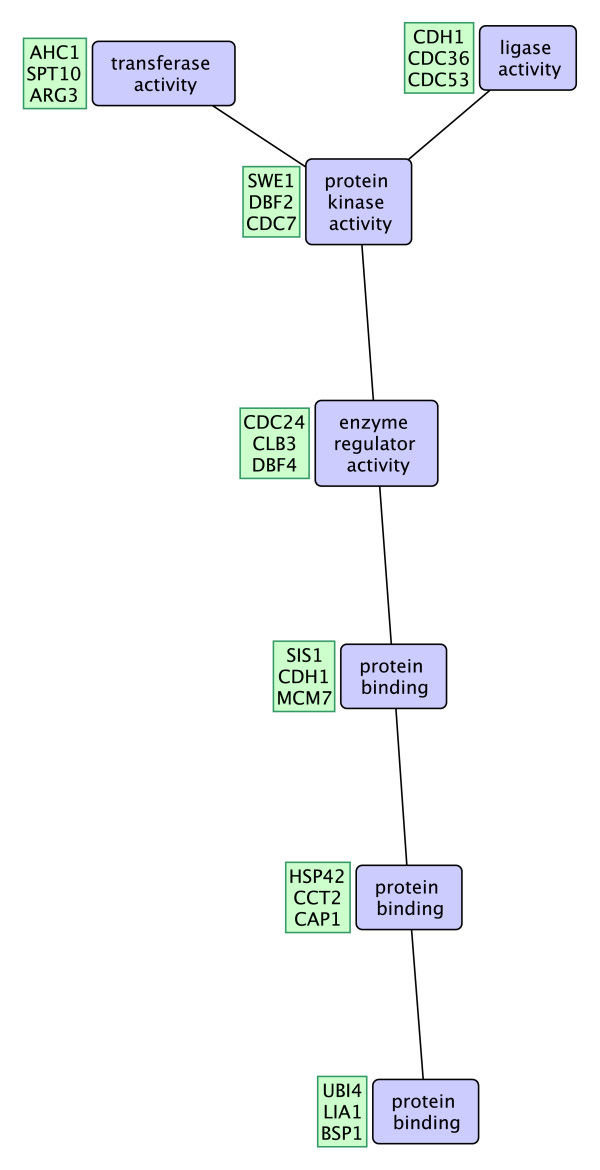
**A frequent functional interaction pattern in the WI-PHI network**. A frequent functional interaction pattern in the WI-PHI network. The tree shaped pattern includes 7 proteins and has 15 non-overlapping occurrences in the network (z-score = 7.29).

Larger functional patterns are identified in the WI-PHI network which contains interactions predicted by integration of multiple data sources. Figure 9 shows a frequent functional pattern of 7 functional terms. The pattern is a long linear cascade that branches at the end of the path. The pattern contains proteins from various functional categories: ligase, transferase, kinase, enzyme regulator, and protein binding activities.

**Figure 10:**
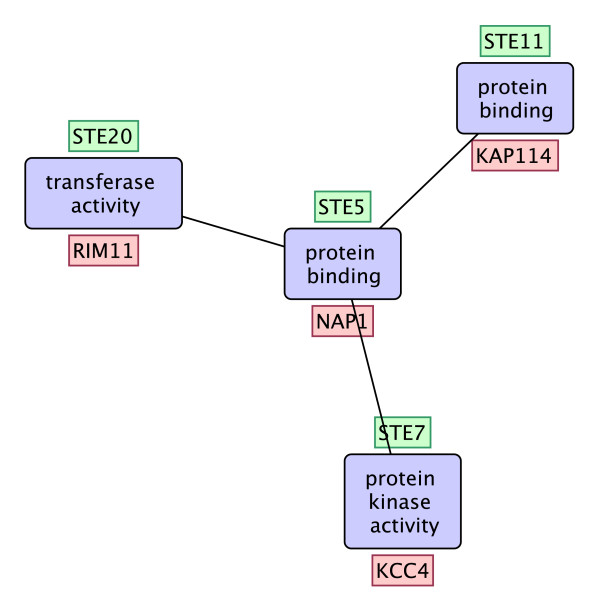
**A functional interaction pattern related to the MAPK signaling pathway**. A frequent functional interaction pattern which has a supporting embedding that completely overlaps with the MAPK signalling pathway.

A frequent functional interaction pattern in the STRING network which has a supporting embedding that completely overlaps with the MAPK signalling pathway is given in Figure 10 (z-score = 3.03). The GO terms of the functional interaction pattern is given inside blue rectangles. The four genes that are members of the MAPK signaling pathway are shown at the top of the nodes in green boxes. This functional interaction pattern has 15 supporting embeddings one of which is shown inside red boxes under the nodes. This particular embedding contains proteins which are not members of any known KEGG pathway. KCC4 is a kinase which coordinate cell cycle progression with the organization of the peripheral cytoskeleton. KCC4 forms a complex with NAP1 and NAP1 interacts with the other two proteins in the functional interaction pattern. This type of analyses allow biologists to study functional interaction patterns that recur in different contexts.

**Figure 11:**
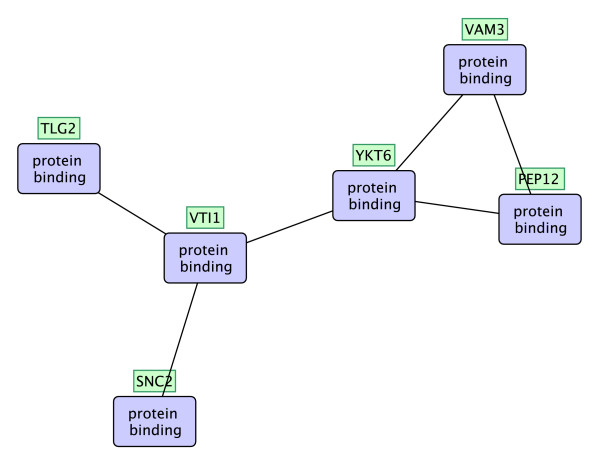
**A functional interaction pattern related to the SNARE interactions in vesicular transport pathway**. A frequent functional interaction pattern which has a supporting embedding that completely overlaps with the SNARE interactions in vesicular transport pathway.

Figure 11 shows another interaction pattern which has a supporting embedding (shown in green rectangles) that completely overlaps with the SNARE interactions in vesicular transport pathway.

Some of the embeddings of the discovered patterns may correspond to previously uncharacterized interaction modules, because the networks we have used are basically results of high-throughput assays. A possible future research direction following-up on our study would be to analyze novel embeddings of the reported patterns by wet-lab experiments and verify them biologically.

### Discussion

In this section, we discuss a number of points that effect the utility of PPISpan and point to other possible applications of PPISpan on protein-protein interaction networks. First of all, the quality of the input PPI network is the most important factor that effects the results of PPISpan pattern search. It is known that current genome-scale protein interaction networks contain considerable amount of false positive interactions and they are far from complete [5]. In order to reduce the effect of noise, we have ran PPISpan on both experimentally determined and predicted PPI networks. A possible follow-up study would be to compare the frequent interaction patterns discovered in different PPI networks.

Note that PPISpan uses a frequent subgraph search heuristic which does not guarantee optimality. Especially, the number of non-overlapping embeddings of a functional interaction pattern may be greater than what is reported by PPISpan if an exhaustive search to find the optimal embeddings is used. PPISpan searches for exact occurrences of patterns in the network; therefore, is bound to overlook interaction patterns with missing edges (i.e., false negatives). On the other hand, false positive interactions are likely to produce interaction patterns which are not observed *in vivo*. An approximate frequent pattern mining algorithm would be ideal for such noisy PPI networks. Another important factor that effects the quality of the detected interaction patterns is the accuracy and specificity of the labels of proteins, i.e., GO annotations. We have not used the electronically inferred annotations to avoid possible additional noise. Node labels are another important aspect that effect the meaning and specificity of the interaction patterns discovered. In this study, we have used the GO Slim Molecular Function ontology which is actually a broad categorization of various molecular functions. This broad categorization produces patterns that are not very specific; hence, it may be difficult to come up with a detailed biological interpretation. However, we provide a framework in which GO annotations at different specificity levels can be used to explore interaction patterns at different levels.

One could also label the proteins in the PPI network with labels other than GO molecular function annotations. For example, using GO cellular component annotations to label the proteins, would be beneficial for finding interaction patterns, e.g., signaling cascades, that span multiple compartments in a cell. Other genome-wide annotations, or protein features can also be used to label the PPI network for mining interaction patterns.

PPISpan can easily be adopted to discover common motifs in multiple organisms. The union graph of multiple GO enriched PPI networks can be given as input to the PPISpan algorithm and each embedding of an interaction pattern can be tagged with the respective organism identifier. The resulting frequent interaction patterns that span multiple organisms can then be identified easily. Since GO annotations are not organism specific, using GO annotations to label the PPI networks would be the ideal choice for this purpose.

## Conclusion

In this article, we proposed a new frequent pattern identification technique, PPISpan, for mining frequent functional interaction patterns in PPI networks. We utilized molecular function Gene Ontology annotations to assign non-unique labels to proteins of a PPI network, and identified significantly frequent functional interaction patterns. We applied PPISpan on experimentally determined and predicted PPI networks of baker's yeast (*Saccharomyces cerevisiae*) labeled with molecular function GO terms and identified a number of potentially interesting patterns. We have identified a number of interesting interaction patterns which offer a new perspective into the modular organization of protein-protein interaction networks. Most of the patterns we found were trees. Cycles were rare. This observation suggests that approximate but fast algorithms for tree pattern mining can be utilized to search for patterns in PPI networks to achieve near interactive response times.

As future work, we plan to search for frequent patterns in protein-protein interaction networks of other organisms such as human [48]. We also plan to investigate "generalized patterns" by deploying relevant techniques previously used for frequent itemset mining [29].

## Methods

### The Datasets

We use three PPI networks of yeast available in public databases. The Database of Interacting Proteins (DIP) [46] (April 11, 2007 version) provides experimental interaction data constructed from high-throughput experiments. The DIP network contains 17,491 interactions for 4,932 proteins. The DIP protein-protein interaction network is represented as an undirected, unweighted graph. We ignore self interactions.

The STRING database contains confidence weighted predicted protein interaction for a number of organisms [49]. We used the top 20050 yeast interactions above the confidence threshold 0.95. The set of interactions covers 2952 proteins in the yeast proteome. Because of the utilized data sources such as gene expression data, the predicted interactions may include indirect interactions apart from physical interactions.

WI-PHI provides a weighted yeast interactome enriched for direct physical interactions [50]. Indirect interactions are minimized in WI-PHI. The complete set of interactions provided by WI-PHI contains 50,000 interacting protein pairs. We have used the first 20097 interactions with weight > 9.4183 in order to have a network with a comparable size to DIP and STRING PPI networks.

We have used the Gene Ontology annotations to assign functional category labels to the proteins of the PPI network. The Gene Ontology (GO) project is a collaborative effort to address the need for consistent descriptions of gene products in different databases. The three main categories in GO provide descriptions for biological processes, cellular components, and molecular functions in a species-independent manner. The hierarchical structure of GO allows annotators to assign properties to gene products at different levels, depending on how much is known about a gene product. In this study, we use the GO Slim terms (see Table 1) of the molecular function category of the Gene Ontology, with the purpose of labeling the proteins of a PPI network with broad functional categories, such as transcription factors and kinases. Our goal is to identify significantly frequent interaction patterns involving proteins of certain functions and occurring in different contexts in the PPI network. A protein is allowed to have multiple labels and all possible combinations are tested when a node of a pattern is to be matched with a protein in the network. In this study, we use the *Saccharomyces cerevisiae *GO annotations downloaded from the GO web site on November 5, 2007. GO Slim mappings of the annotations are obtained by following the parent links of annotated GO terms until a GO Slim term is reached.

### PPISpan Algorithm

Numerous algorithms have been developed for discovering frequent patterns in graphs [21,31,34,42-45]. Most of the algorithms follow two basic steps: candidate generation and frequency counting. In the "candidate generation" step, all possible patterns are enumerated, and later in the "frequency counting" step, each candidate pattern is validated by counting its embeddings in the whole graph. If the count (also called the support of the pattern) is above a certain threshold then the pattern is considered frequent. Counting the frequency of a candidate pattern in a large graph (e.g., a genome-scale protein interaction network) requires the use of subgraph isomorphism test which is known to be NP-complete [51-53]. Therefore, most algorithms aim at reducing the number of candidate patterns by identifying and eliminating the redundant ones. gSpan by Yan and Han [31] achieves this by computing a depth-first search based canonical labeling of candidate patterns and pruning the search space when identical labelings are found.

In order to decide whether a subgraph is frequent or not, Kuramochi and Karypis [43] use approximate Maximum Independent Set algorithms and find whether the overlap graph of a subgraph's non-identical embeddings contain an independent set whose size is above a given threshold. They experimented with real data sets from different domains including protein interaction networks with about 20,000 vertices. They were able to detect frequent patterns of up to 8 vertices in the PPI network. However, their main objective was to test the running time of the algorithm on an undirected network of uniquely identified nodes; hence, they did not report any biologically interesting interaction patterns.

Hu *et al*. developed an algorithm, CODENSE [21], to mine recurrent patterns across large collections of genome-wide networks. They applied CODENSE to discover coherent clusters across 39 co-expression networks and used homogenous clusters for functional annotation of uncharacterized genes. The uncharacterized genes in a cluster are annotated with the functional category of the most significantly expressed GO term in that cluster. You *et al*. propose a graph based data mining tool, SUBDUE [45], which is used to better understand KEGG metabolic pathways and find biologically meaningful patterns. The patterns are used to distinguish pathways, or provide the common features in several pathways. Koyuturk *et al*. [42] proposes an algorithm for mining KEGG metabolic pathways based on frequent itemset mining. It takes advantage of the sparse nature of metabolic pathways to reduce the associated computational cost. Later in another study [34], they make use of the fact that there exist many proteins in an organism that are orthologous to each other. Orthologous nodes in the graph dataset are contracted into single nodes; and hence, the underlying isomorphism problem is considerably simplified.

We modified the gSpan algorithm [31] to better suit for GO annotated genome-scale protein-protein interaction networks. gSpan generates candidate patterns from a Depth First Search (DFS) Code Tree where a node in the tree represents a single candidate. Each time, a new candidate is generated and then is tested for support. If a pattern does not have enough support, its children in the DFS Code tree are ignored. Similarly, if the DFS Code of a candidate is minimum (meaning that candidate graph is isomorphic to a candidate graph processed earlier), then its children in the DFS Code tree are ignored. These two pruning techniques makes gSpan very efficient and adaptable for application to different types of networks including protein interaction networks.

gSpan implicitly assumes that minimum depth-first search (DFS) code computation of a candidate is less costly than frequency counting of itself and its descendants combined. This is usually not true in our setting especially when the average node per label is low and when we are merely interested in finding highest frequent patterns (See Results section). As the gSpan algorithm delves deeper into the lower levels of the DFS Code Tree, the minimum DFS calculation gets extremely harder while the cost of support computation stays practically constant. Since this support computation is very likely to fail (i.e., pruning false positives), the total computational cost of pruning false positives amounts less than the cost of minimum DFS code calculation. Therefore, we use a lightweight feasibility function to decide whether support computation for a pattern is more likely to cost less than computing the minimum DFS code, and skip the latter depending on the output of this function.

In this study, we define a novel lexicographical ordering of edge and vertex labels to speed up the overall search for frequent patterns in a protein-protein interaction network. The ordering of vertices is based on the number of appearances (frequency) of each vertex label in the network. This is in descending order, i.e., the more frequent label precedes the less frequent label. Similarly, we define a frequency based ordering for edges. An edge is represented by a pair of vertex labels and a label pair with low frequency precedes the one with higher frequency. gSpan algorithm removes an edge from the graph after it finishes searching the DFS Code Tree rooted at that edge. Therefore removing the less frequent edges from the PPI network in the early stages of the search, later help reduce the time for pruning false positives for more frequent edges. Similar to CloseGraph [54], we also modified gSpan to only output the maximal patterns, where a maximal pattern is a frequent subgraph which is not a proper subgraph of any other frequent graph. The PPISpan algorithm is given below in two parts: 1) Algorithm *PPISpan *– the main iteration over each edge in the PPI network, 2) Algorithm *Subgraphs *– the module which extends each subgraph into larger subgraphs.

**Algorithm: **PPISpan (*G*, *L*, *minSup*)

**1: **Set the vertex labels in *G *with GO terms from the desired GO level *L*

**2: ***S *← all frequent 1-edge graphs in *G *in frequency based lexicographical order

**3: **for each edge *e *∈ *S *(in ascending frequency order) do

**4: **   SubGraphs(*e*, *minSup*, *e*)

**5: **   remove *e *from *G*

**Algorithm: **SubGraphs(*s*, *minSup*, *ext*)

**1: **if (feasible(*s*, *ext*))

**2: **   if DFS code of *s *is not equal to its minimum DFS code

**3: **      return

**4: ***C *← Generate all children of *s *(by growing an edge, *ext*)

**5: ***maximal *← **true**

**6: **for each *c *∈ *C *(in DFS lexicographical order) do

**7: **   if support(*c*) ≥ *minSup*

**8: **      SubGraphs(*c*, *minSup*, *c.ext*)

**9: **      *maximal *← **false**

**10: **if (*maximal*)

**11: **   output *s*

As gSpan's graph growth in the DFS Code Tree dictates, a child pattern is one edge different than the parent. Therefore, the embeddings of the parent may be used to compute the embeddings of the child. An embedding of a pattern is a subgraph in the large input graph such that it is isomorphic to the pattern. We store the embeddings of a parent pattern graph in order to use it for the child pattern's support computation. The support computation of child pattern *c *of *s *in Line 7 of the SubGraph algorithm is carried out by using the embeddings of *s*. We define the support of a pattern *p *as the number of non-overlapping embeddings of *p *in the network. The exact location of each embedding and complete mapping between the vertices of the pattern and the vertices of embedding is stored along with the pattern. These stored embeddings make the subgraph matching task significantly simpler and quicker because the graph matching operations are not repeated for the child once they have been completed for the parent. We defined a Boolean feasibility function of *s *and *ext *such that the function returns true if frequency of *ext *is greater than or equal to the mean frequency of edges in *s *plus the standard deviation of frequency of edges in *s*. In other words, if the frequency of *ext *in the network is one standard deviation greater than or equal to the frequencies of edges in *s *then the pattern *s *is considered feasible and its minimum DFS code is computed. Otherwise, this computation is skipped.

### Statistical Significance of a Frequent Pattern

In order to provide a global measure to compare patterns of different sizes, we compute the statistical significance of a frequent pattern in addition to the support of the pattern. We compute Bonferroni corrected z-score of a pattern by counting similar patterns (with at least the same size as the observed pattern) in 100 different random networks. The random networks are generated such that they have the same degree and functional annotation distribution as the original PPI network. The z-score is given by the distance (in number of standard deviations) between the support of the pattern in the original network and the average support of similar patterns in the ensemble of random networks. Bonferroni correction is applied after z-scores of all frequent patterns are computed.

## Authors' contributions

MET designed and implemented PPISpan, and ran the experiments on the *Saccharomyces cerevisiae *DIP network. TC ran the experiments on the STRING and WI-PHI networks. TC designed and ran the MIPS and KEGG comparison experiments. TC supervised the study and interpreted the significant patterns biologically. All authors read and approved the final manuscript.

## References

[B1] Uetz P, Giot L, Cagney G, Mansfield TA, Judson RS, Knight JR, Lockshon D, Narayan V, Srinivasan M, Pochart P, Qureshi-Emili A, Li Y, Godwin B, Conover D, Kalbfleisch T, Vijayadamodar G, Yang M, Johnston M, Fields S, Rothberg JM (2000). A comprehensive analysis of protein-protein interactions in Saccharomyces cerevisiae. Nature.

[B2] Ito T, Chiba T, Ozawa R, Yoshida M, Hattori M, Sakaki Y (2001). A comprehensive two-hybrid analysis to explore the yeast protein interactome. Proceedings of the National Academy of Sciences.

[B3] Gavin A, Bosche M, Krause R, Grandi P, Marzioch M, Bauer A, Schultz J, Rick J, Michon A, Cruciat C, Remor M, Höfert C, Schelder M, Brajenovic M, Ruffner H, Merino A, Klein K, Hudak M, Dickson D, Rudi T, Gnau V, Bauch A, Bastuck S, Huhse B, Leutwein C, Heurtier M, Copley R, Edelmann A, Querfurth E, Rybin V, Drewes G, Raida M, Bouwmeester T, Bork P, Seraphin B, Kuster B, Neubauer G, Superti-Furga G (2002). Functional organization of the yeast proteome by systematic analysis of protein complexes. Nature.

[B4] Ho Y, Gruhler A, Heilbut A, Bader GD, Moore L, Adams SL, Millar A, Taylor P, Bennett K, Boutilier K, Yang L, Wolting C, Donaldson I, Schandorff S, Shewnarane J, Vo M, Taggart J, Goudreault M, Muskat B, Alfarano C, Dewar D, Lin Z, Michalickova K, Willems AR, Sassi H, Nielsen PA, Rasmussen KJ, Andersen JR, Johansen LE, Hansen LH, Jespersen H, Podtelejnikov A, Nielsen E, Crawford J, Poulsen V, Sørensen BD, Matthiesen J, Hendrickson RC, Gleeson F, Pawson T, Moran MF, Durocher D, Mann M, Hogue CWV, Figeys D, Tyers M (2002). Systematic identification of protein complexes in Saccharomyces cerevisiae by mass spectrometry. Nature.

[B5] von Mering C, Krause R, Snel B, Cornell M, Oliver SG, Fields S, Bork P (2002). Comparative assessment of large-scale data sets of protein-protein interactions. Nature.

[B6] Jansen R, Yu H, Greenbaum D, Kluger Y, Krogan NJ, Chung S, Emili A, Snyder M, Greenblatt JF, Gerstein M (2003). A bayesian networks approach for predicting protein-protein interactions from genomic data. Science.

[B7] Lee I, Date SV, Adai AT, Marcotte EM (2004). A probabilistic functional network of yeast genes. Science.

[B8] Przulj N, Wigle D, Jurisica I (2004). Functional topology in a network of protein interactions. Bioinformatics.

[B9] Valente AXCN, Cusick ME (2006). Yeast Protein Interactome topology provides framework for coordinated-functionality. Nucl Acids Res.

[B10] Luo F, Yang Y, Chen CF, Chang R, Zhou J, Scheuermann RH (2007). Modular organization of protein interaction networks. Bioinformatics.

[B11] Sharan R, Suthram S, Kelley RM, Kuhn T, McCuine S, Uetz P, Sittler T, Karp RM, Ideker T (2005). From the Cover: Conserved patterns of protein interaction in multiple species. Proceedings of the National Academy of Sciences.

[B12] Hirsh E, Sharan R (2007). Identification of conserved protein complexes based on a model of protein network evolution. Bioinformatics.

[B13] Bader G, Hogue C (2003). An automated method for finding molecular complexes in large protein interaction networks. BMC Bioinformatics.

[B14] Spirin V, Mirny LA (2003). Protein complexes and functional modules in molecular networks. Proceedings of the National Academy of Sciences.

[B15] Asthana S, King OD, Gibbons FD, Roth FP (2004). Predicting protein complex membership using probabilistic network reliability. Genome Res.

[B16] Scott J, Ideker T, Karp RM, Sharan R (2005). Efficient algorithms for detecting signaling pathways in protein interaction networks. RECOMB.

[B17] Brohee S, van Helden J (2006). Evaluation of clustering algorithms for protein-protein interaction networks. BMC Bioinformatics.

[B18] Cakmak A, Ozsoyoglu G (2007). Mining biological networks for unknown pathways. Bioinformatics.

[B19] Letovsky S, Kasif S (2003). Predicting protein function from protein/protein interaction data: a probabilistic approach. Bioinformatics.

[B20] Lanckriet GRG, Deng M, Cristianini N, Jordan MI, Noble WS (2004). Kernel-based data fusion and its application to protein function prediction in yeast. Proceedings of the Pacific Symposium on Biocomputing.

[B21] Hu H, Yan X, Huang Y, Han J, Zhou XJ (2005). Mining coherent dense subgraphs across massive biological networks for functional discovery. Bioinformatics.

[B22] Patil A, Nakamura H (2005). Filtering high-throughput protein-protein interaction data using a combination of genomic features. BMC Bioinformatics.

[B23] Chen J, Hsu W, Lee ML, Ng SK (2006). Increasing confidence of protein interactomes using network topological metrics. Bioinformatics.

[B24] Suthram S, Shlomi T, Ruppin E, Sharan R, Ideker T (2006). A direct comparison of protein interaction confidence assignment schemes. BMC Bioinformatics.

[B25] Collins SR, Kemmeren P, Zhao XC, Greenblatt JF, Spencer F, Holstege FC, Weissman JS, Krogan NJ (2007). Toward a comprehensive atlas of the physical interactome of Saccharomyces cerevisiae. Mol Cell Proteomics.

[B26] Mahdavi M, Lin YH (2007). False positive reduction in protein-protein interaction predictions using gene ontology annotations. BMC Bioinformatics.

[B27] Kashtan N, Itzkovitz S, Milo R, Alon U (2002). Mfinder tool guide.

[B28] Yeger-Lotem E, Sattath S, Kashtan N, Itzkovitz S, Milo R, Pinter RY, Alon U, Margalit H (2004). Network motifs in integrated cellular networks of transcription-regulation and protein-protein interaction. Proceedings of the National Academy of Sciences.

[B29] Inokuchi A (2004). Mining generalized substructures from a set of labeled graphs. ICDM '04: Proceedings of the Fourth IEEE International Conference on Data Mining (ICDM'04).

[B30] Nijssen S, Kok JN (2005). The gaston tool for frequent subgraph mining. Electronic Notes in Theoretical Computer Science.

[B31] Yan X, Han J (2002). gSpan: graph-based substructure pattern mining. ICDM '02: Proceedings of the 2002 IEEE International Conference on Data Mining (ICDM'02).

[B32] Oyama T, Kitano K, Satou K, Ito T (2002). Extraction of knowledge on protein-protein interaction by association rule discovery. Bioinformatics.

[B33] Besemann C, Denton A, Yekkirala A (2004). Differential association rule mining for the study of protein-protein interaction networks. BIOKDD04: 4th Workshop on Data Mining in Bioinformatics (with SIGKDD Conference).

[B34] Koyuturk M, Kim Y, Subramaniam S, Szpankowski W, Grama A (2006). Detecting conserved interaction patterns in biological networks. Journal of Computational Biology.

[B35] The Gene Ontology Consortium (2000). Gene Ontology: tool for the unification of biology. Nature Genet.

[B36] GO slim and subset guide. http://www.geneontology.org/GO.slims.shtml.

[B37] Kanehisa M, Goto S (2000). KEGG: Kyoto Encyclopedia of Genes and Genomes. Nucl Acids Res.

[B38] Pandey J, Koyuturk M, Kim Y, Szpankowski W, Subramaniam S, Grama A (2007). Functional annotation of regulatory pathways. Bioinformatics.

[B39] Pandey J, Koyutürk M, Szpankowski W, Grama A (2008). Annotating pathways of interaction networks. Proceedings of the Pacific Symposium on Biocomputing.

[B40] Levy ED, Pereira-Leal JB, Chothia C, Teichmann SA (2006). 3D complex: a structural classification of protein complexes. PLoS Computational Biology.

[B41] Bernard A, Vaughn DS, Hartemink AJ, Speed TP, Huang H (2007). Reconstructing the topology of protein complexes. RECOMB, Volume 4453 of Lecture Notes in Computer Science Springer.

[B42] Koyutürk M, Grama A, Szpankowski W (2004). An efficient algorithm for detecting frequent subgraphs in biological networks. Bioinformatics.

[B43] Kuramochi M, Karypis G (2005). Finding frequent patterns in a large sparse graph. Data Min Knowl Discov.

[B44] Wernicke S (2005). A faster algorithm for detecting network motifs. WABI.

[B45] You CH, Holder LB, Cook DJ (2006). Application of graph-based data mining to metabolic pathways. ICDMW '06: Proceedings of the Sixth IEEE International Conference on Data Mining – Workshops.

[B46] Salwinski L, Miller CS, Smith AJ, Pettit FK, Bowie JU, Eisenberg D (2004). The Database of Interacting Proteins: 2004 update. Nucl Acids Res.

[B47] Mewes HW, Amid C, Arnold R, Frishman D, Guldener U, Mannhaupt G, Munsterkotter M, Pagel P, Strack N, Stumpflen V, Warfsmann J, Ruepp A (2004). MIPS: analysis and annotation of proteins from whole genomes. Nucleic Acids Research.

[B48] Mathivanan S, Periaswamy B, Gandhi T, Kandasamy K, Suresh S, Mohmood R, Ramachandra Y, Pandey A (2006). An evaluation of human protein-protein interaction data in the public domain. BMC Bioinformatics.

[B49] von Mering C, Jensen LJ, Kuhn M, Chaffron S, Doerks T, Kruger B, Snel B, Bork P (2007). STRING 7-recent developments in the integration and prediction of protein interactions. Nucleic Acids Research.

[B50] Kiemer L, Costa S, Ueffing M, Cesareni G (2007). WI-PHI: A weighted yeast interactome enriched for direct physical interactions. PROTEOMICS.

[B51] Cordella LP, Foggia P, Sansone C, Vento M (2004). A (sub)graph isomorphism algorithm for matching large graphs. IEEE Trans Pattern Anal Mach Intell.

[B52] McKay BD (1981). Practical graph isomorphism. Congressus Numerantium.

[B53] Ullmann JR (1976). An algorithm for subgraph isomorphism. J ACM.

[B54] Yan X, Han J (2003). CloseGraph: mining closed frequent graph patterns. KDD '03: Proceedings of the ninth ACM SIGKDD international conference on Knowledge discovery and data mining.

